# Aging Like Milk or Fine Wine: Content Analysis of Reactions to One’s Future Self on TikTok

**DOI:** 10.2196/70794

**Published:** 2026-08-18

**Authors:** Reuben Ng, Nicole Indran

**Affiliations:** 1 Lee Kuan Yew School of Public Policy National University of Singapore Singapore Singapore

**Keywords:** aging, TikTok, social media, artificial intelligence, AI, ageism, stigma, cultural beliefs

## Abstract

**Background:**

The “aged” filter has gone viral on TikTok. This filter leverages AI to superimpose signs of aging, such as wrinkles, crow’s feet, and sagging skin, onto a user’s face. Notably, several dermatologists have attested to the accuracy of this filter in simulating the effects of aging. Reactions among the TikTok community have been mixed, with some utterly dismayed but others wholeheartedly embracing the sight of their aged visages.

**Objective:**

This study takes a deep dive into the ways in which TikTok users react to their aged selves through a content analysis of videos featuring the aged filter. Understanding how they respond to the filter can shed light on the types of meanings commonly attributed to old age as a social construct. The following research questions form the premise of our content analysis of videos on TikTok: (1) How do individuals react to the way their possible future selves look? (2) What types of attitudes toward old age are being conveyed in these videos? (3) What do these responses say about prevailing cultural beliefs, values, and attitudes toward old age?

**Methods:**

To build a comprehensive dataset, we compiled all publicly available videos (N=2682) using the following hashtags as search queries: “#AgedFilter,” “#OldFilter,” and “#AgeFilter.” Collectively, these hashtags amassed >2,068,100,000 views at the time of writing this manuscript. After applying our exclusion criteria, the dataset comprised 681 videos, which received a total of 34,382,236 likes, 1,204,741 favorites, and 211,006 comments. Both deductive and inductive approaches informed our content analysis.

**Results:**

A total of 7 themes emerged from our content analysis. Half of the videos (n=342, 50%) fell under theme 1, “anxiety about aging.” The second-largest theme (n=95, 14%) focused on the “pursuit of youthfulness” (theme 2). Theme 3 was about “aging with confidence” (n=84, 12%) and theme 4 was about the “beauty of aging” (n=54, 8%). Theme 5 examined the “privilege of aging” (n=48, 7%) and theme 6 examined “generational connections” (n=43, 6%). Theme 7 covered the “inevitability of aging” (n=15, 2%).

**Conclusions:**

This study marks one of the first attempts to analyze videos using the aged filter on TikTok. Our findings suggest that ageist beliefs may be prevalent among some users. Going forward, there is value in adopting a definition of beauty that transcends age. By doing so, aging can be reframed as a natural part of life to be embraced rather than as a grim reality to be dreaded.

## Introduction

TikTok boasts a presence in more than 160 countries and lays claim to approximately a billion monthly active users [1]. One defining characteristic that sets TikTok apart from other social media services is its extensive array of creative tools—filters, sounds, and stickers, among others—which users can experiment with when creating videos. In July 2023, the “aged” filter went viral on TikTok [2]. This filter essentially leverages AI to superimpose signs of aging, such as wrinkles, crow’s feet, and sagging skin, onto a user’s face. Notably, several dermatologists have attested to the accuracy of this filter in simulating the effects of aging [2]. Reactions among the TikTok community have been mixed, with some utterly dismayed and others wholeheartedly embracing the sight of their aged visages [2]. This study takes a deep dive into the ways TikTok users react to their aged selves through a content analysis of videos using the aged filter.

From a conceptual viewpoint, this study marks one of the first attempts to analyze videos using the aged filter on TikTok. Other gerontological studies that have dealt with social media focus on other topics such as stereotypes about older adults [3-7]. Understanding how individuals respond to the aged filter can shed light on their attitudes toward the physical changes that attend the aging process. From a practical viewpoint, this study can illuminate the types of meanings people attribute to old age as a social construct. It is well documented that TikTok is populated primarily by younger individuals. On the basis of data from July 2023, more than 37% of TikTok’s global user base is composed of individuals aged 18 to 24 years. Those aged 25 to 34 years accounted for approximately 32% of the TikTok populace [8]. Insights from this study can support broader efforts to improve societal perceptions of old age.

In accordance with the theory of social constructionism, all human knowledge is produced, communicated, and sustained in social contexts [9]. To conceive of age as a social construct is to acknowledge that although aging itself is a biological fact, the meanings assigned to it are by no means cast in stone [10]. Rather, these meanings are colored by the values, attitudes, and narratives that prevail in society at a particular time [10].

In virtually all contemporary societies, standards of attractiveness are predicated on having a youthful appearance [11-13]. Specifically, youth has been constructed as the epitome of health, vitality, and beauty. In contrast, aging is typically associated with a loss of appearance-related assets, especially in terms of social and sexual desirability [11,14]. Perhaps the phenomenon that best exemplifies the hegemonic ideal of youthfulness is the enduring profitability of the antiaging industry, which purveys a plenitude of products, regimens, and services often peddled as elixirs of youth [15,16]. The global antiaging market was valued at approximately US $58.5 billion in 2020 and is forecast to experience a compound annual growth rate of 7% from 2021 to 2026 [17].

Critical feminists have censured the antiaging industry for the way it has problematized a natural transition, cast old age as the enemy, and ultimately engineered a climate of fear surrounding the aging process [18]. Many of them have argued that the construct of antiaging is a capitalistic framework designed to cash in on feelings of insecurity [19]. These feelings of insecurity are implanted through marketing that casts wrinkles and fine lines as issues to be “treated” or “prevented,” and aging as a process to be “delayed,” “repaired,” or “inhibited” [20]. A striking example of how the antiaging industry instills a fear of aging is the way Elizabeth Arden advertised its PREVAGE moisturizer. Specifically, a fencing mask was used to symbolize the protective nature of the brand’s antiaging skincare technology. One of the lines in the advertisement, “[p]repare yourself for the beauty battlefield,” further underscores the message that aging ought to be fought and conquered [21].

Another camp emphasizes the need to pay attention to the role of agency when assessing one’s reasons for conducting antiaging work. Some postulate that this qualifies as disidentification [22,23], an adaptive strategy that involves a reconceptualization of the self in a way that minimizes one’s aging identity as a basis of self-evaluation. More precisely, in a society where beauty is frequently entwined with youthfulness, partaking in antiaging work can serve as a way to elevate one’s self-esteem and confidence [24]. Research states that cosmetic treatments can enhance psychological well-being and ameliorate appearance-based distress [25]. Engaging in antiaging work may also function as a way to sidestep the marginalization that ensues when one is visibly older [12,26].

It is widely accepted that a sense of anxiety accompanies the notion of aging [27-29]. Lasher and Faulkender [30] define this anxiety as the anticipation of losses that aging brings about. They consider that anxiety about aging encompasses 4 distinct dimensions: physical, psychological, social, and transpersonal. Particularly apposite to this study is the physical dimension, which includes factors such as perceptions of one’s health status, concerns about physical self-efficacy, as well as the physical changes that come with growing older. Anxiety about aging may manifest in 3 types of fears: fear of aging, fear of being old, and fear of older people [30]. Anxiety about aging is said to be intertwined with anxiety about death [31,32].

The perceived loss of physical attractiveness can pose a threat to one’s self-esteem and psychological well-being, especially in a world where good looks constitute a form of social currency [33]. Additionally, as per the theory of stereotype embodiment [34], the negativity ascribed to societal meanings of old age can be hazardous to one’s health. When assimilated as part of one’s culture, age stereotypes may eventually become self-definitions that shape health outcomes in later life. Since the 1990s, research done in regions including America, Europe, and Asia has found that older adults who hold negative age stereotypes have a lower sense of efficacy, a higher risk of depression, and poorer cardiovascular health [34-36]. Conversely, those who embrace positive age stereotypes generally enjoy better functional health, enhanced well-being, and increased longevity [34,37,38]. There is also evidence that people who endorse negative age stereotypes in their younger years confront a greater likelihood of experiencing a cardiovascular event in later life than those who hold positive age stereotypes [34].

The following research questions form the premise of our content analysis of videos on TikTok: (1) How do individuals react to the way their possible future selves look? (2) What types of attitudes toward old age are being conveyed in these videos? (3) What do these responses say about prevailing cultural beliefs, values, and attitudes toward old age?

## Methods

### Dataset

Consistent with previous research [39], a new TikTok (ByteDance Ltd) account was created to compile the videos. This was done to reduce bias since videos on the app are arranged based on a complex algorithm, which takes into account the popularity of the video (measured by views, likes, comments, and shares), the popularity of the creator (measured by followers and engagement), any previous content that was engaged with, and the geographic location of the device where the app was accessed [39]. No content was previously engaged with to ensure a regular user’s experience when using the app [39].

The aged filter on TikTok is a type of visual effect designed to simulate an aged appearance. Several versions of this filter exist on the platform, but they all serve the same purpose. We compiled all publicly available videos (N=2682) using the following hashtags as search queries: “#AgedFilter” (n=901), “#OldFilter” (n=877), and “#AgeFilter” (n=904). Hashtags enhance the searchability of content by organizing it into categories. Collectively, these hashtags amassed >2,068,100,000 views at the time of writing this manuscript. Videos were compiled from August 21, 2023, to September 21, 2023. We removed (1) videos where the filter served purposes other than the self-examination of one’s future appearance, such as role-playing or performing skits (n=745); (2) videos not in English (n=682); (3) videos where the filter was applied to participants other than the user, such as celebrities or animals (n=246); (4) videos with a different filter or without a filter (n=218); (5) videos where the user had no discernible reaction (n=90); and (6) videos that appeared more than once in the dataset, in which case only one was retained (n=20). After applying the exclusion criteria, our dataset consisted of 681 videos, which received a total of 34,382,236 likes, 1,204,741 favorites, and 211,006 comments. Table 1 shows the data collection process.

**Table 1:** Themes identified in TikTok content featuring the aged filter based on a content analysis of 681 videos.

Themes	Videos, n (%)
Anxiety about aging	342 (50)
Pursuit of youthfulness	95 (14)
Aging with confidence	84 (12)
Beauty of aging	54 (8)
Privilege of aging	48 (7)
Generational connections	43 (6)
Inevitability of aging	15 (2)

### Content Coding of TikTok Videos

Following past research [40], the coding rubric was designed using both deductive and inductive modes of reasoning [41]. Analyses led by a directed or deductive approach begin with the identification of an initial set of codes based on past scholarship [41]. Meanwhile, in inductive content analyses, codes are derived directly from the data [41]. We used both deductive and inductive approaches to ensure certain pertinent assumptions informed the analysis, even as we were mindful that new categories would emerge inductively.

To develop a preliminary codebook, we identified categories based on scholarship concerning perceptions of physical appearance in later life [12,14,33]. All the videos were viewed twice by the authors to ensure familiarity with the data. The objective of the first reading was to ascertain the validity of the initial set of categories, as well as to modify the codebook until all variables were refined and defined clearly. During this first reading, a new category was added whenever a video featured a particular attribute that could not be suitably coded into any of the existing categories and appeared frequently enough to warrant its own category. The objective of the second reading was to ensure we had a framework sufficiently representative of the different types of videos to finalize the coding rubric.

To maintain analytic rigor, the 2 coders had regular discussions during which any discrepancies were reviewed and adjudicated. Areas of major overlap were identified and sectioned into broader themes. The percentage agreement between the 2 raters was 96.5% with a weighted Cohen κ of 0.93 (*P*<.001), showing high interrater reliability. Seven themes emerged from this iterative procedure. It must be highlighted that a video may be classified under more than one theme. As mentioned in previous research, categories in a content analysis need not be mutually exclusive, although they should be internally homogeneous (ie, coherent within themes) and externally heterogeneous (ie, distinct from each other) as far as possible [42,43].

### Ethical Considerations

This study analyzed publicly available TikTok data and did not involve interaction with human participants. According to institutional guidelines, ethical review board approval was not required for this study. Informed consent was not obtained as all data were publicly accessible and anonymized prior to analysis.

## Results

### Summary of Insights From Content Analysis of TikTok Videos

Of the 681 videos, half (n=342, 50%) fell under theme 1, “anxiety about aging.” The “pursuit of youthfulness” was the focal point of theme 2 (n=95, 14%). Theme 3 was about “aging with confidence” (n=84, 12%), while theme 4 was about the “beauty of aging” (n=54, 8%). The “privilege of aging” was discussed in 7% (n=48) of the videos. Theme 6, “generational connections,” made up 6% (n=43) of the dataset. Theme 7 covered the “inevitability of aging” (n=15, 2%). Table 1 summarizes the themes.

### Theme 1: Anxiety About Aging

In this theme, users reacted to their aged selves with varying levels of displeasure. “Shocked,” “traumatized,” “disturbed,” “depressed,” “devastated,” “terrified,” and “enraged” are but some of the words TikTokers used upon seeing themselves through the filter. Many were convinced the filter did them “dirty.” One said the filter “lower[ed] [her] self-esteem,” and another asserted that the creator of the filter essentially “amplified her body dysmorphia.” An audio clip used by multiple users featured a woman uttering, “I need to buy a gun.”

Some evoked death-related imagery to describe their future appearances. For instance, one TikToker likened herself to someone who had “just been dug up from the grave.” Another bewailed, “It looks like I am [expletive] decaying, bro. I don’t even look human, bro, I look like a [expletive] corpse, bro.” One insisted he looked like he was “two seconds away from [his] final breath.”

Comparisons to movie characters were commonplace. One referred to herself as a “wrinkly Voldemort,” and another as the “after version of Rose from Titanic.” One poster claimed she looked like “something from The Conjuring.”

References to witches emerged in a couple of uploads. A case in point is a video where a user asked, “Why do I look like the witch in the [expletive] Bog that steals your firstborn child? Why do I look like I put a curse on the princess in the castle?” Another opined that she bore a resemblance to a “witch living in the woods.”

There were posters who took issue with how fellow users seemed to age “well” or “gracefully.” In particular, one individual lamented, “Some people age like fine wine, some people age like milk. I am of the milk variety.”

Many were vexed by how old they appeared. “I look like I fought in World War II,” exclaimed one user. Another griped, “I look like I am 110 years old and was left out in the sun for an amount of time.” One user groaned, “I look like someone left me in the pool for ages.”

A few TikTokers took a more dramatic approach, declaring their intent to withdraw from the aging process altogether. Statements that encapsulated this sentiment include “Old isn’t for me, guys. Peace out,” “So, youth is not in my future,” “I need to die young,” and “I will not be partaking in aging.”

Some were discomfited by how unapproachable or unfriendly they appeared. For instance, one exclaimed, “I look like I eat children.” Another complained that she looked like “she would bite the nursing home staff.”

### Theme 2: Pursuit of Youthfulness

Posts under theme 2 featured users attempting to stave off the aging process through enhanced skincare regimens, cosmetic procedures, or antiaging supplements. Such videos typically depicted individuals frantically applying sunscreen or moisturizer, occasionally using copious amounts for dramatic effect. Similar videos showcased users incorporating facial rollers into their skincare routines.

Many users conveyed a desire to undergo cosmetic procedures such as Botox (AbbVie Inc) or face-lifts. “Give me the botox right now. I want whatever Kris Jenner has.... Before you start commenting ‘aging is beautiful’, shut the [expletive] up, okay, this is not. This is hideous,” commanded one user. Another video contained a text sticker stating, “Running to put on liquid botox and sunscreen [because] I refuse to believe this is what I will look like when I’m old.”

One TikToker reached for a tub of collagen peptides and other supplements, vowing to “prove the filter wrong.” Another shared that she would be “switching from 0.05% to 0.1% tretinoin” in order not to “age like spoiled milk.”

### Theme 3: Aging With Confidence

Videos in this category featured users feeling content or confident with their older selves. Some enthused over how “sophisticated” and “powerful” they looked. One effused unapologetically, “I’m gonna be such a hot granny.” Another was certain of her ability to continue “luring men” in her advanced age. One proudly declared he was “aging like fine wine” and dubbed himself a “good-looking man.” There was even a playful warning from one poster to “hide the abuelas,” presumably because of his attractiveness. Several TikTokers called themselves “cute.” For example, one commented, “I hope I age this good. I look like a cute old lady.”

### Theme 4: Beauty of Aging

This theme revolved around the positive aspects of aging, focusing on the personal growth and enrichment it brings.

Several users embraced aging as a path toward increased wisdom, inner strength, and personal fulfillment. “I can’t wait to grow old...to have experienced so much life, hold powerful wisdom, and witness myself blossom and change over time. I get so emotional and filled with love seeing the older version of me,” one remarked. Another shared, “In my family, wrinkles show laughter and grey hair means wisdom. Age is beauty. I’m so excited to meet her. She looks like she gives the best hugs and runs the local crafts store.” One believed that her older self appeared fulfilled, affirming, “She looks like she’s lived.” Another individual volunteered that being able to reach old age means having “lived and survived.”

A group of posters dwelled on the warmth radiated by their future selves. Some relished the prospect of becoming grandparents. One user mentioned that she looked like “she gives the best hugs, is loved by all the grandkids, and always slips you money every time she sees you.” Another focused on the pleasant demeanor of her aged self, saying, “I like her. She has kind eyes. She’s lived a full life. She’s the type of woman who will comfort you when you’re sad. She deeply understands people.”

### Theme 5: Privilege of Aging

In these posts, users reflected on aging as a “gift,” “blessing,” or “privilege.” A fair share of individuals spoke about how their struggles with mental illness made them grateful for the opportunity to envision life in the future. For example, one confessed, “I’ve been so mentally ill my whole life. I’ve genuinely never pictured living this long before.” Similarly, a self-proclaimed cancer survivor announced that it would be the “greatest gift” to live till old age. A paramedic said the filter made him think about “all the people [he] was not able to save,” believing these people would have desired to live a long life. He concluded, “Aging is a gift. It’s a gift to be able to lead a fulfilled, long life.”

Some TikTokers were disheartened by the overwhelming negativity surrounding the filter. One maintained that aging is a “privilege,” not a “punishment.” Another criticized the TikTok community for being “wildly ageist,” stressing that anyone would be “lucky to live this long.”

### Theme 6: Generational Connections

Videos under theme 6 had users reveling in the fact that their aged selves were the spitting image of their parents or grandparents. A representative video contained a caption that read, “What’s so wonderful about this is I immediately see my grandma, dad, mum, aunties, and uncles in my features. We are combinations of so many people.”

A handful of posters had a deep sense of admiration for their parents or grandparents, which in turn fueled their excitement about growing older. For instance, one gushed, “I look like my grandma who I think is the most beautiful woman.” Another anticipated “aging gracefully” like her grandmother. This sentiment was especially pronounced for those who had lost their parents or grandparents.

### Theme 7: Inevitability of Aging

In this theme, users either normalized aging as a natural part of life or resigned themselves to the inevitability of its implications. One TikToker spoke reassuringly, “You’re gonna age and that’s okay.” Another put forth, “We all have to get old one day.”

One individual was upfront about becoming less attractive. “I wanna embrace the fact that we’re all gonna look like this someday and nobody’s gonna die looking sexy,” she proclaimed.

## Discussion

### Principal Findings

In this study, we examined the reactions of individuals to the appearance of their possible future selves by performing a content analysis of videos using the aged filter on TikTok. Our analysis unveiled 7 themes: “anxiety about aging” (theme 1), “pursuit of youthfulness” (theme 2), “aging with confidence” (theme 3), “beauty of aging” (theme 4), “privilege of aging” (theme 5), “generational connections” (theme 6), and “inevitability of aging” (theme 7).

Collectively, themes 1 and 2 accounted for more than two-thirds of the videos, which underscores the widespread negativity surrounding the concept of aging among users. In particular, old age is perceived as entailing a loss of one’s physical attractiveness [14,30]. An outsized number of users couched their future selves in words that were highly pejorative. Many also conveyed their intent to pay more attention to their skincare routines or to undergo cosmetic procedures to prolong their youthful countenance and quell the anxiety of growing older. These themes reflect deeply ingrained beliefs that equate youth with beauty and old age with undesirability. Such beliefs can exact a toll on one’s self-esteem and overall psychological well-being [33]. Furthermore, through the lens of the theory of stereotype embodiment, these negative beliefs about aging can jeopardize one’s overall health outcomes [34,37,38].

Theme 3 portrayed users with a positive self-image and sense of self-assurance regarding growing older, attributes that will likely stand them in good stead as they eventually advance into later life. Posts under this theme offer a powerful and refreshing counternarrative that reimagines old age as a period characterized by continued appeal. However, this sense of contentment or empowerment could very well be tethered to the perception of one’s physical appearance. One person commented that he aged like “fine wine,” insinuating that less appealing ways to age do exist. It is possible that their reactions would be less favorable had the filter presented a version of themselves with more pronounced signs of aging.

Posts under theme 4 reflect the belief that old age is a phase marked by the accrual of experience and wisdom, as well as the development of a mellower personality. Implicit in these posts is a recognition that aging is a multifaceted journey that goes beyond mere physical transformation. In particular, growing older involves certain emotional, social, and psychological changes [44]. Similarly, theme 5 illustrates that for some members of the TikTok population, the experience of aging is not so much a matter of one’s changing visage as it is an opportunity to enjoy a long and fulfilling life. Both themes 4 and 5 substantiate findings showing that as people age, their priorities may evolve such that appearance no longer figures into their self-evaluations as prominently [45-47].

Videos under theme 6 depicted users getting emotional over the striking resemblance between their future selves and their parents or grandparents. A growing body of evidence documents that many individuals tend to perceive their grandparents more positively than the general older populace [48,49]. Moreover, being close to one’s grandparents has been linked to more positive views of older adults [50,51]. The excitement expressed by certain individuals about being able to look like their older family members may be anchored in strong emotional ties.

Theme 7 demonstrated users having come to terms with the reality of the aging process. Cognizant of the fact that they would eventually deviate from the hegemonic ideal of youthfulness, some TikTokers adopted cognitive strategies that involved adjusting their expectations about their eventual selves [47]. For instance, a user immediately reconciled herself to the fact that she would no longer be considered attractive by societal standards, thereby decoupling her self-worth from youth-based ideals and accepting her older self.

### Implications

Our findings suggest multiple avenues for combating ageism. First, there is a need to step up efforts to reframe aging. Over the years, various social movements, such as the “body positivity” movement, have sprouted up to counter prevailing beauty standards deemed harmful. The principles of inclusivity underlying these movements could be extended to the way society perceives old age. Recently, a number of beauty companies have eschewed the term “antiaging.” Instead, these companies now opt for seemingly anodyne alternatives, such as “regeneration,” “renewal,” and “vitality,” which, at the end of the day, however, continue to promulgate the idea that aging skin is undesirable [52]. True inclusivity could involve accepting natural signs of aging. Even as some may rely on antiaging solutions as a way to cope with the aging process [12,24,26], the reality is that all individuals will eventually reach a point where physical signs of aging can no longer be camouflaged by cosmetic interventions [45].

Second, given its preponderant role in stigmatizing old age, the media bears significant responsibility for improving portrayals of older adults [12]. Proactive steps could be taken to increase their representation. For example, media outlets and advertising agencies could actively include older individuals in beauty-related content. This may help transmit the message that beauty is not the prerogative of youth and that aging is but a natural and inevitable part of life. It could also foster positive age stereotypes and lead to more favorable health outcomes [34].

Third, it is paramount that society unlearns the notion that aging brings with it a loss of physical attractiveness. Instead, aging should be recognized as a time of increased experience [53] and emotional intelligence [54]. More importantly, it should be seen as a privilege since not all individuals will live to see old age [55]. Promoting the idea of self-love and self-acceptance may be worthwhile as well [56].

Fourth, tapping into the deep emotional bonds that people share with their parents or grandparents may inspire a more positive outlook on aging [48,49]. When individuals discern aspects of their family members in themselves, they may navigate their own aging journey more favorably. Policymakers could involve grandparents or aging parents in interventions to combat ageism [57,58], harnessing them as proof that aging can indeed be a time of continued growth.

Finally, our findings make a case for implementing initiatives to reframe aging from an early age so that individuals are exposed to positive narratives on aging long before they reach later life. To this end, efforts to reframe aging could be integrated into schools and workplaces.

### Limitations and Future Directions

This study is subject to several limitations. First, our findings are unique to a segment of TikTok users whose videos happened to be included in this study and are therefore not generalizable to the wider population. Second, as we did not have access to the motivations that drove users to create the videos, our interpretations were molded by our own thoughts and assumptions. It is worth noting that TikTok is a platform that thrives on entertaining content. Hence, some may have reacted in specific ways to bolster engagement. Third, we lacked information on the demographic characteristics of users, such as their gender. We also refrained from using automated recognition systems given their potential to perpetuate gender biases [59]. Thus, we could not comment on how demographic factors influence individuals’ attitudes toward their older selves. It is well established that men and women are subject to different sets of expectations as far as aging is concerned [11,60]. This double standard of aging places women in a position where they contend with greater losses as growing older is often perceived to entail an erosion of their most valued social asset: their physical allure. Men, in contrast, are sometimes regarded as more attractive as they age [61]. Fourth, a substantial number of videos had to be excluded as they were not in English. Future studies could examine this topic in other cultural contexts [62]. Finally, although we recorded the overall number of videos for each theme, we did not track the number of videos associated with the various subthemes. Future research could adopt a quantitative approach [63] by noting down the number of videos for different subthemes. This would lay the groundwork for a regression analysis to explore potential relationships in the data [64, 65].

Other avenues for further gerontological inquiry include an investigation of the psychological effects of using the aged filter. In addition, there is room for an analysis of how reactions to one’s aged self differ across age groups. Studying the comments section of videos that use the aged filter presents another promising direction for subsequent research.

### Conclusions

This study has shed light on the prevalence of ageist beliefs among some users of TikTok. Going forward, there is value in adopting a definition of beauty that transcends age. By doing so, aging can be reframed as a natural part of life to be embraced rather than as a grim reality to be dreaded.

## Data Availability

Data are publicly available on TikTok [66].
